# Supplementary material for: Cochlear transcript diversity and its role in auditory functions implied by an otoferlin short isoform
Source: Nat Commun. 2023 May 29;14:3085. doi: 10.1038/s41467-023-38621-3 (PMC10227054; doi:10.1038/s41467-023-38621-3)
Supplement: Supplementary file 1 — Supplemental Information [file 41467_2023_38621_MOESM1_ESM.pdf]

Supplemental Information:

Cochlear transcript diversity and its role in auditory functions implicated by an otoferlin short isoform

Huihui Liu[1,2,3†], Hongchao Liu[1,2,3†], Longhao Wang[1,2,3†], Lei Song[1,2, 3†] , Guixian Jiang [1,2,3†], Qing Lu[1,2,3,4,] Tao Yang[1,2,3], Hu Peng[5], Ruijie Cai[1,2,3], Xingle Zhao[1,2,3], Ting Zhao[1,2,3], Hao Wu[1,2,3*]

[1]Department of Otolaryngology-Head and Neck Surgery, Shanghai Ninth People's Hospital, Shanghai Jiao Tong University School of Medicine, Shanghai, China 200011;

[2]Ear Institute, Shanghai Jiao Tong University School of Medicine, Shanghai, China 200011;

[3]Shanghai Key Laboratory of Translational Medicine on Ear and Nose Diseases, Shanghai, China 200011;

[4]Key Laboratory for the Genetics of Developmental and Neuropsychiatric Disorders, Ministry of Education, Bio-X Institutes, Shanghai Jiao Tong University, Shanghai, China 200240.

[5]Department of Otolaryngology-Head and Neck Surgery, Changzheng Hospital, Second Military Medical University, Shanghai, China 200003

CONTENTS:

Supplementary Fig. 1 is related to Figure 1. Quality control metrics for ScISOr-Seq data.

Supplementary Fig. 2 is related to Figure 3. Identification of alternative splicing events and protein diversity.

Supplementary Fig. 3 is related to Figure 4. The diversity of transcripts for deafness genes and identified an inner ear-specific *Otof* isoform.

Supplementary Fig. 4 is related to Figure 4. The expression of the otoferlin short isoform in the inner ear.

Supplementary Fig. 5 is related to Figure 4. *Otof-ΔC* mice exhibited a decreased ABR

wave I amplitude with normal latency and the heterozygous *Otof-ΔC* and *Otof* $^{-/-}$ mice showed normal ABR.

Supplementary Fig. 6 is related to Figure 4. The inner hair cell ribbon synapse counts.

Supplementary Fig. 7 is related to Figure 4. *Otof-ΔC* IHCs showed normal Ca^{2+} influx but reduced presynaptic exocytosis under different depolarization amplitudes.

Supplementary Fig. 8 is related to Figure 5. Decreased cytosolic synaptic vesicles, accumulation of coated structures, and ELVs in *Otof-ΔC* IHCs.

Supplementary Fig. 9 is related to Figure 6. Protein binding assay of the otoferlin canonical and short isoform.

Supplementary Data 1: Differentially Expressed genes for all cell clusters (Illumina).

Supplementary Data 2: Differentially Expressed genes for all cell clusters (PacBio).

Supplementary Data 3: The isoform characters of the detected genes using PacBio Iso-seq.

Supplementary Data 4: Isoforms in each cell type.

Supplementary Data 5: The primers for RT-PCR and the sequences from Sanger Sequence.

Supplementary Data 6: Detected peptides from MS-based proteomics and the cell type specific peptide.

Supplementary Data 7: ScISOr-Seq specific novel peptides.

UCSC Genes track: UCSC Genes track file can be uploaded in the UCSC Genome Browser (https://genome-asia.ucsc.edu/) and manipulated to view the novel isoforms in different cell types.
