# Supplementary material for: Cochlear transcript diversity and its role in auditory functions implied by an otoferlin short isoform
Source: Nat Commun. 2023 May 29;14:3085. doi: 10.1038/s41467-023-38621-3 (PMC10227054; doi:10.1038/s41467-023-38621-3)
Supplement: Supplementary file 3 — Description of Additional Supplementary Files [file 41467_2023_38621_MOESM3_ESM.pdf]

**Description of Additional Supplementary Files**

File Name: Supplementary Data 1

Description: Differentially Expressed genes for all cell clusters (Illumina).

File Name: Supplementary Data 2

Description: Differentially Expressed genes for all cell clusters (PacBio).

File Name: Supplementary Data 3

Description: The isoform characters of the detected genes using PacBio Iso-seq.

File Name: Supplementary Data 4

Description: Isoforms in each cell type.

File Name: Supplementary Data 5

Description: The primers for RT-PCR and the sequences from Sanger Sequence.

File Name: Supplementary Data 6

Description: Detected peptides from MS-based proteomics and the cell type specific peptide.

File Name: Supplementary Data 7

Description: ScISOr-Seq specific novel peptides

File Name: UCSC Genes track

Description: UCSC Genes track file can be uploaded in the UCSC Genome Browser ( https://genome-asia.ucsc.edu/) and manipulated to view the novel isoforms in different cell types.
