# Supplementary material for: Cochlear transcript diversity and its role in auditory functions implied by an otoferlin short isoform
Source: Nat Commun. 2023 May 29;14:3085. doi: 10.1038/s41467-023-38621-3 (PMC10227054; doi:10.1038/s41467-023-38621-3)
Supplement: Supplementary file 11 — Reporting Summary [file 41467_2023_38621_MOESM11_ESM.pdf]

nature portfolio

Corresponding author(s): Hao Wu

Last updated by author(s): Apr 26, 2023

Reporting Summary

Nature Portfolio wishes to improve the reproducibility of the work that we publish. This form provides structure for consistency and transparency in reporting. For further information on Nature Portfolio policies, see our Editorial Policies and the Editorial Policy Checklist.

Statistics

For all statistical analyses, confirm that the following items are present in the figure legend, table legend, main text, or Methods section.

n/a	Confirmed	
☐	☒	The exact sample size (*n*) for each experimental group/condition, given as a discrete number and unit of measurement
☐	☒	A statement on whether measurements were taken from distinct samples or whether the same sample was measured repeatedly
☐	☒	The statistical test(s) used AND whether they are one- or two-sided<br>*Only common tests should be described solely by name; describe more complex techniques in the Methods section.*
☒	☐	A description of all covariates tested
☐	☒	A description of any assumptions or corrections, such as tests of normality and adjustment for multiple comparisons
☐	☒	A full description of the statistical parameters including central tendency (e.g. means) or other basic estimates (e.g. regression coefficient) AND variation (e.g. standard deviation) or associated estimates of uncertainty (e.g. confidence intervals)
☐	☒	For null hypothesis testing, the test statistic (e.g. *F*, *t*, *r*) with confidence intervals, effect sizes, degrees of freedom and *P* value noted<br>*Give P values as exact values whenever suitable.*
☒	☐	For Bayesian analysis, information on the choice of priors and Markov chain Monte Carlo settings
☒	☐	For hierarchical and complex designs, identification of the appropriate level for tests and full reporting of outcomes
☐	☒	Estimates of effect sizes (e.g. Cohen's *d*, Pearson's *r*), indicating how they were calculated

Our web collection on statistics for biologists contains articles on many of the points above.

Software and code

Policy information about availability of computer code

Data collection	ScRNA-Seq was performed on Illumina NovaSeq 6000 platform, PacBio data were done using PacBio Sequel II platform, Oxford Nanopore sequencing data were collected on PromethION platforms, and the Bulk RNA-seq was performed using BGISEQ-500 sequencing technology. Mass spectrometry-based proteomics was performed on an EASY-nanoLC 1200 system (Thermo Fisher Scientific) connected to an Orbitrap Fusion Lumos Tribrid mass spectrometer (Thermo Fisher Scientific). Confocal imaging data was collected using Zeiss LSM880 with Zen imaging software (v2.3). TEM data was collected using Orius 1200A camera with a digital micrograph software package (v 01.12) on JEOL JEM-1011 transmission electron microscope. Protein binding assay signals were visualized using enhanced chemiluminescence solutions (180-5001, Tanon) and the Tanon 5200 Imaging System (Tanon). For ABRs and IHCs patch clamping recordings, TDT workstation(Tucker-Davis Technologies) with BioSigRZ (v5.1) and EPC10/2 amplifier (HEKA Electronics) driven by Patchmaster software (v2 x 90.2) were used to collect data respectively.
Data analysis	RStudio(v 1.3.1056) with R (v 4.2.2), Seurat(v 4.3.0), SMRT Link (v 8.0), Lima tools(v 1.10.0), cDNA_Cupcake(v 8.2), Isoseq3-refine (v 3.2.2), Minimap2 (v 2.2), SQANTI3 (v 4.0), GeneMarkS-T (v 5.1), SUPPA2(v 2.3), LIGER package (v 1.0.0), IGV (v 2.11.1), StringTie2 (v2.1.4), GffCompare (v 0.12.6), SnapGene software (v 4.2.4) and STAR (v2.7.9a) were used for ScRNA-Seq, PacBio data, ONT data, RNA-seq data and sanger sequencing analysis. Spectronaut (v 14) was used for mass spectrometry-based proteomics data analysis.<br>Analysis of confocal image were performed using Imaris (v 9.2.0),GraphPad Prism (v 8.0),and Igor Pro (v 7.0.4.1) were used for data quantification and statistical analysis.

For manuscripts utilizing custom algorithms or software that are central to the research but not yet described in published literature, software must be made available to editors and reviewers. We strongly encourage code deposition in a community repository (e.g. GitHub). See the Nature Portfolio guidelines for submitting code & software for further information.

Data

Policy information about availability of data

All manuscripts must include a data availability statement. This statement should provide the following information, where applicable:

- Accession codes, unique identifiers, or web links for publicly available datasets
- A description of any restrictions on data availability
- For clinical datasets or third party data, please ensure that the statement adheres to our policy

The raw data of scRNA-seq, Iso-seq and bulk RNA-seq generated in this study have been deposited in the NCBI Sequence Read Archive (SRA) under accession BioProject codes: PRJNA759047 (BioSample: SAMN21155185). Mass spectrometry-based proteomics data have been deposited at the ProteomeXchange Consortium via the PRIDE partner repository with the accession number PXD031062. RNA-seq data of mouse cerebral cortex tissue and hippocampus tissue are available in a public repository from the https://www.encodeproject.org/ website (ENCODE: ENCSR248KDJ and ENCSR330DDD). The reference mouse genome (GRCm38/mm10) was downloaded from the UCSC genome Browser (http://genome-asia.ucsc.edu/cgi-bin/hgGateway?redirect=manual&source=genome.ucsc.edu). scRNA-seq data can be accessed at https://umgear.org/index.html?multigene_plots=0&layout_id=0d6584b2&gene_symbol_exact_match=1&gene_symbol=ocm. The novel transcripts in different cell types can be accessed using the UCSC Genes track file at https://genome-asia.ucsc.edu/. The remaining data were provided with this paper within the Article, Supplementary Information or Source Data file. Source data and the UCSC Genes track file are provided in this paper.

Human research participants

Policy information about studies involving human research participants and Sex and Gender in Research.

Reporting on sex and gender	n/a
Population characteristics	n/a
Recruitment	n/a
Ethics oversight	n/a

Note that full information on the approval of the study protocol must also be provided in the manuscript.

Field-specific reporting

Please select the one below that is the best fit for your research. If you are not sure, read the appropriate sections before making your selection.

☒ Life sciences ☐ Behavioural & social sciences ☐ Ecological, evolutionary & environmental sciences

For a reference copy of the document with all sections, see nature.com/documents/nr-reporting-summary-flat.pdf

Life sciences study design

All studies must disclose on these points even when the disclosure is negative.

Sample size	No statistical methods were used to predetermine sample size estimates. Sample size was determined based on standards in the field and experiments to obtain statistical significance and reproducibility. At least n=3 mice/cell of each group were used to meet the minimal requirements for statistical analysis and the detailed sample size was demonstrated in the figure, figure legends or the source data file.
Data exclusions	ScRNA-seq: Standard data preprocessing was performed, with the following genes and cells excluded from downstream analyses: i) genes expressed in fewer than ten cells (i.e., extremely weakly expressed genes); ii) cells with <200 unique genes and 1,500 UMIs, indicating lowquality cells or empty droplets; iii) cells with >3,000 unique genes and 15,000 UMIs, indicating cell doublets or multiplets; iv) cells with >5% mitochondrial genes or >5% stress genes (i.e., potentially low-quality or dying cells).<br>Iso-seq: data was excluded without identified cDNA primers and poly(A) tails.<br>For other animal experiments, no data related to the manuscript were excluded.
Replication	To ensure the replications of the findings, the in vivo and in vitro experiments were repeated at least three times as indicated in the figure legends. All experimental data was reliably reproduced in multiple independent experiments. The sample size were used to ensure reproducibility are declared in the figure, figure legends or the source data file.
Randomization	Randomization for mouse works was achieved by distributing experimental mice (Otof-ΔC, Otof-ΔS, Otof-full KO or wild type mouse) into different cages (the cages did not indicate wild type and genotyping mice).
Blinding	Investigators were blinded for data acquisition and data analysis.

Reporting for specific materials, systems and methods

We require information from authors about some types of materials, experimental systems and methods used in many studies. Here, indicate whether each material, system or method listed is relevant to your study. If you are not sure if a list item applies to your research, read the appropriate section before selecting a response.

Materials & experimental systems		
n/a	Involved in the study	
☐	☒	Antibodies
☐	☒	Eukaryotic cell lines
☒	☐	Palaeontology and archaeology
☐	☒	Animals and other organisms
☒	☐	Clinical data
☒	☐	Dual use research of concern

Methods		
n/a	Involved in the study	
☒	☐	ChIP-seq
☒	☐	Flow cytometry
☒	☐	MRI-based neuroimaging

Antibodies

Antibodies used

The following antibodies were used in this study:
Mouse anti-CtBP2 antibody (BD Biosciences,catalog#: 612044,dilution:1:200);
Mouse anti-GluR2 antibody (Merck-Millipore, catalog#: MAB397,dilution:1:200);
Mouse anti-Otoferlin antibody (Abeam,catalog#: ab53233, dilution:1:200);
Rabbit anti-Otoferlin antibody (Synaptic Systems,catalog#: 178003, dilution:1:200);
Mouse anti- Myosin-VIIa DSHB, USA, no: 138-1, dilution:1:200)
Rabbit anti-Endophilin 1 antibody (Synaptic Systems,catalog#: 159002, dilution:1:200)
Alexa Fluor 568-conjugated goat anti-mouse IgG1 (Invitrogen, USA, no: A-21124, dilution:1:500)
Alexa Fluor 647-conjugated goat anti-mouse IgG2a (Invitrogen, USA, no: A-21241, dilution:1:500)
Alexa Fluor 488-conjugated goat anti-rabbit IgG (Invitrogen, USA, no: A-11008, dilution:1:500)
Mouse anti-GFP tag (Proteintech, USA, no: 66002-1-Ig, dilution:1:2000)
Rabbit anti-HA-Tag (Cell Signaling Technology, USA, no: 3724T, dilution: 1:1000)
Mouse anti- 6xHis-tag (Sigma, USA, no: H1029, dilution: 1:3000)
Mouse anti-GST (Santa Cruz Biotechnology, USA, no: sc-138, dilution: 1:500).
Peroxidase AffiniPure Goat Anti-Mouse IgG (H+L) (Jackson ImmunoResearch, USA, no: 115-035-003, dilution: 1:10000)
Peroxidase AffiniPure Goat Anti-Rabbit IgG (H+L) (Jackson ImmunoResearch, USA, no: 115-035-003, dilution: 1:10000)

Validation

All primary antibodies (commercial) were used according to manufacturer's instructions in this study and at least preliminary testing in our laboratories. Precise information on the validation of each antibody for species and applications can be found in the following links:
Mouse anti-CtBP2 lgGl: https://www.bdbiosciences.com/en-us/products/reagents/microscopy-imaging-reagents/immunofluorescence-reagents/purified-mouse-anti-ctbp2.612044
Mouse anti-GluR2 lgG2a: https://www.merckmillipore.com/CN/zh/product/Anti-Glutamate-Receptor-2-Antibody-extracellularclone-6C4,MM_NF-MAB397
Mouse anti-otoferlin: https://www.abcam.com/products/primary-antibodies/otoferlin-antibody-13a9-ab53233.html
Rabbit anti-otoferlin: https://sysy.com/product/178003#list
Mouse anti-Myosin-Vlla: https://dshb.biology.uiowa.edu/MYO7A-138-1
Rabbit anti-Endophilin 1: https://sysy.com/product/159002
Mouse anti-GFP tag :https://www.ptgcn.com/products/eGFP-Antibody-66002-1-Ig.htm
Rabbit anti-HA-Tag:https://www.cellsignal.com/products/primary-antibodies/ha-tag-c29f4-rabbit-mab/3724?site-search-type=Products&N=4294956287&Ntt=3724t&fromPage=plp&_requestid=3283362
Mouse anti- 6xHis-tag: https://www.sigmaaldrich.com/US/en/product/sigma/h1029
Mouse anti-GST:https://www.scbt.com/p/gst-antibody-b-14?requestFrom=search

Eukaryotic cell lines

Policy information about cell lines and Sex and Gender in Research

Cell line source(s)	HEK-293T cells were purchased from American Type Culture Collection (ATCC).
Authentication	Cells were authenticated using the short tandem repeat (STR) method.
Mycoplasma contamination	Cells tested negative for mycoplasma contamination.
Commonly misidentified lines (See ICLAC register)	No commonly misidentified lines were used.

Animals and other research organisms

Policy information about studies involving animals; ARRIVE guidelines recommended for reporting animal research, and Sex and Gender in Research

Laboratory animals	The following strains of mice were involved in the study:<br>- Wild type mice (C57BL/6J background). Either sex was used for experiments. Experiments were performed at ages of P7, P26-30, and P210.<br>- Otof- ΔC mice (C57BL/6J background). Either sex was used for experiments. Experiments were performed at ages of P26-30.<br>- Otof- ΔS mice (C57BL/6J background). Either sex was used for experiments. Experiments were performed at ages of P26-30.<br>- Otof- full KO mice (C57BL/6J background). Either sex was used for experiments. Experiments were performed at ages of P26-30.<br>Mice were housed for the duration of these experiments in the animal care facility of the Ear Institute of Shanghai Ninth People's Hospital, in affiliation with the Shanghai Jiao Tong University School of Medicine. Mice were maintained in a dark/light cycle of 12 hours / 12 hours. Animals were kept at room temperature with a range of 22-25 ? with humidity of the animal room ranging between 50-60%.
Wild animals	The study did not involve wild animals.
Reporting on sex	We did not observe sex-based differences from our work. Both sexes were used in this study.
Field-collected samples	The study did not involve field-collected samples.
Ethics oversight	The experimental protocol was approved by the Institutional Animal Care and Use Committee at Ninth hospital, School of Medicine, Shanghai Jiao Tong University (SH9H-2022-A926-1) and followed the guidelines for the Care and Use of Laboratory Animals (8th edition), published by the National Institutes of Health (Bethesda, MD, USA).

Note that full information on the approval of the study protocol must also be provided in the manuscript.
