# Supplementary material for: Cochlear transcript diversity and its role in auditory functions implied by an otoferlin short isoform
Source: Nat Commun. 2023 May 29;14:3085. doi: 10.1038/s41467-023-38621-3 (PMC10227054; doi:10.1038/s41467-023-38621-3)
Supplement: Supplementary file 12 — Supplementary Figures [file 41467_2023_38621_MOESM12_ESM.pdf]

Supplementary Figures to

Cochlear transcript diversity and its role in auditory functions implied by an otoferlin short isoform

Supplementary Fig. 1

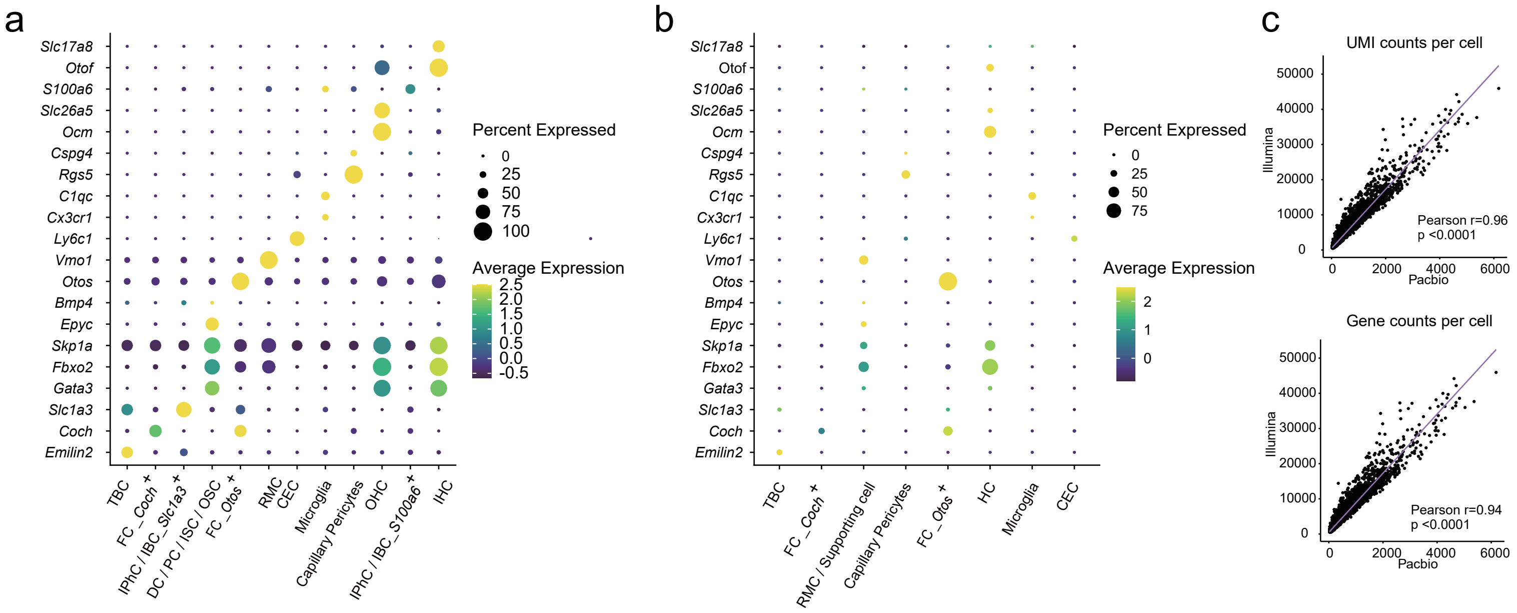

Supplementary Fig. 1 Quality control metrics for ScISOr-Seq data.

a, b Dot plot of average expression and cellular detection rate of representative canonical marker genes across different cell types of the Illumina sequencing data (**a**) and PacBio sequencing data (**b**). **c** The correlation between Illumina and PacBio data showed high reproducibility of both UMIs and genes detected per cell. The Pearson *r* and *p* values (two-tailed *t* test) are shown in each panel.

Supplementary Fig. 2

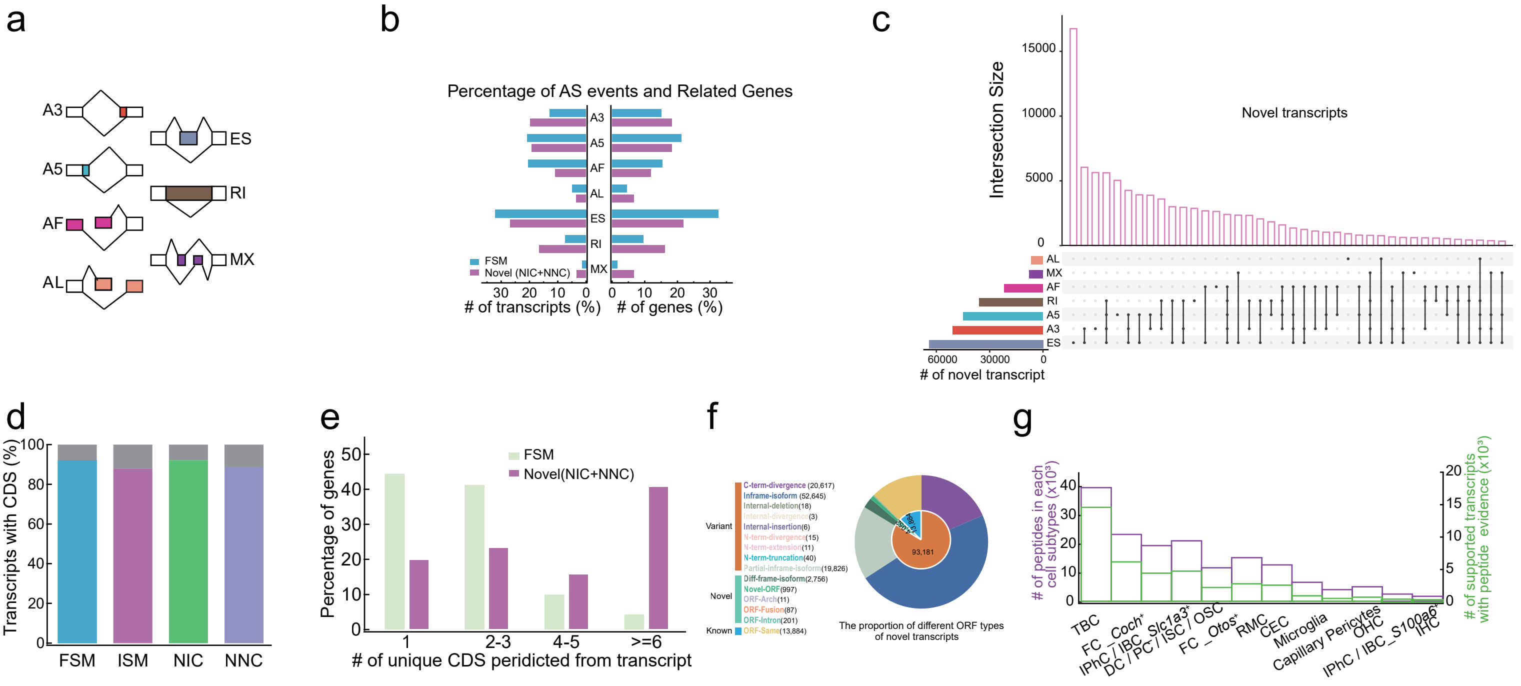

Supplementary Fig. 2 Identification of alternative splicing events and protein diversity.
a Visualization of the seven types of AS events: alternative 3′/ 5′-donor or acceptor (A3/5), alternative first exon (AF), alternative last (AL), exon skipping (ES), retained intron (RI), and mutually exclusive exon (MX). **b** Distributions of AS events and involved genes in known (FSM) and novel (NIC, NNC) transcripts. **c** UpSet plot of interactions among the seven types of novel transcript-related alternative splicing events. One novel transcript may have multiple types of AS events. **d** Percentage of predicted coding sequences (CDSs) from ScISOr-Seq in different categories of transcript isoforms. **e** The number of unique ORFs from novel transcripts per gene revealed that one gene could encode multiple proteins. **f** Pie charts showing the proportion of different ORF types predicted from novel transcripts indicating the contribution to protein diversity. **g** Bar plots revealed the number of detected peptides (left) and supported ORFs (right) with peptide evidence in each cell subtype.

Supplementary Fig. 3

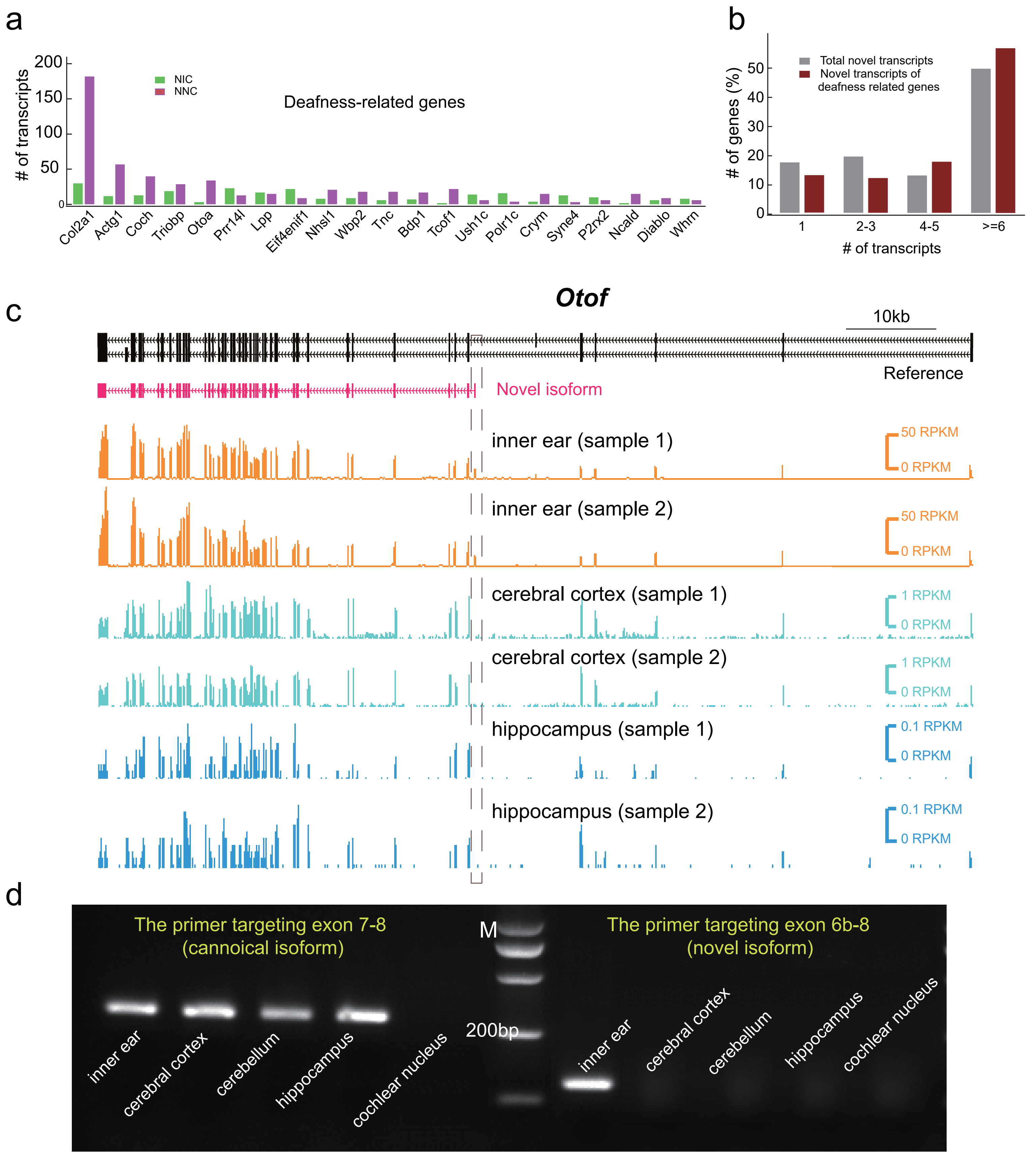

Supplementary Fig. 3 The diversity of transcripts for deafness genes and identified an inner ear-specific *Otof* isoform.

a Total number of novel transcripts cataloged for each deafness-related gene detected by ScISOr-Seq. **b** The number of novel isoforms detected per deafness gene showed higher isoform diversity than those in the cochlea.

c, d A novel *Otof* transcript transcribed from exon 6b was found. Transcript maps of *Otof* isoforms from the mouse inner ear, cerebral cortex, and hippocampus using short-read data were verified by RT–PCR in different tissues (primers targeting 6b-8, and 7-8, respectively). The gel bands figure shows the existence of *Otof* and its novel isoform in different tissues, highlighting inner ear-specific characteristics (representative results of at least three biological replicates are shown).

Supplementary Fig. 4

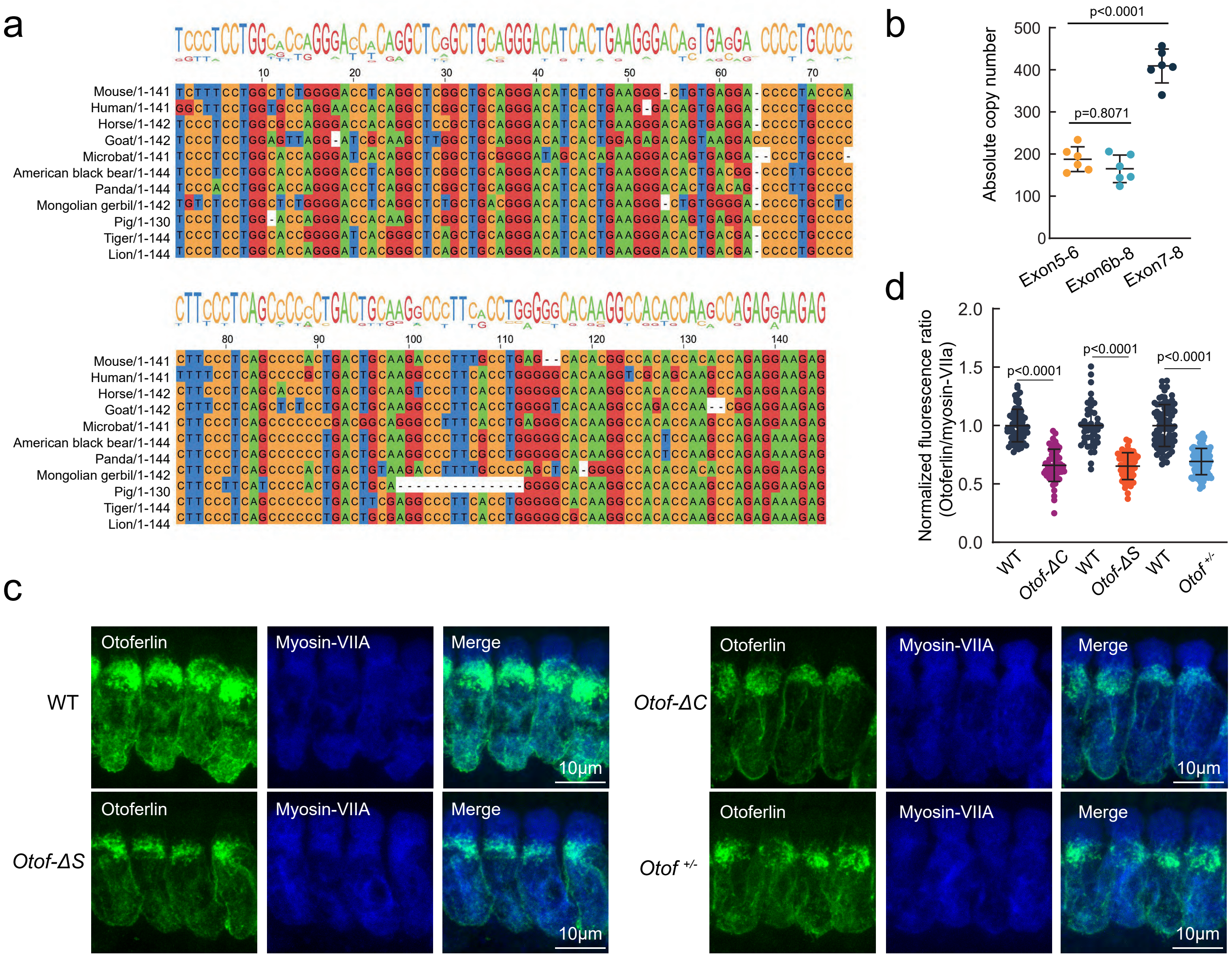

Supplementary Fig. 4 The expression of the otoferlin short isoform in the inner ear.
a Exon 6b was shown to be highly conserved among mammals. **b** The absolute copy numbers of the annotated and unannotated transcripts were comparable, while the expression of both transcripts was higher (one-way ANOVA followed by Bonferroni *post hoc* test). **c, d** Maximum intensity z-projections of confocal sections of IHCs with labeling for otoferlin (targeting the protein fragment encoded by exons 7-8; otoferlin and myosin-VI-IA). Otoferlin immunoreactivity was quantified by normalizing to myosin-VIIA, and the immunofluorescence density ratio was decreased in *Otof-ΔC*, *Otof-ΔS* and *Otof* $^{+/-}$ IHCs. Age-matched and sex-matched littermate WT controls were used for all experiments. Statistical analysis by one-way ANOVA followed by the Bonferroni *post hoc* test with significance indicated (**b**), and two-side unpaired *t* test or Mann-Whitney test with significance indicated (**d**). All data, statistical test used and *p* values can be found in the source data file.

Supplementary Fig. 5

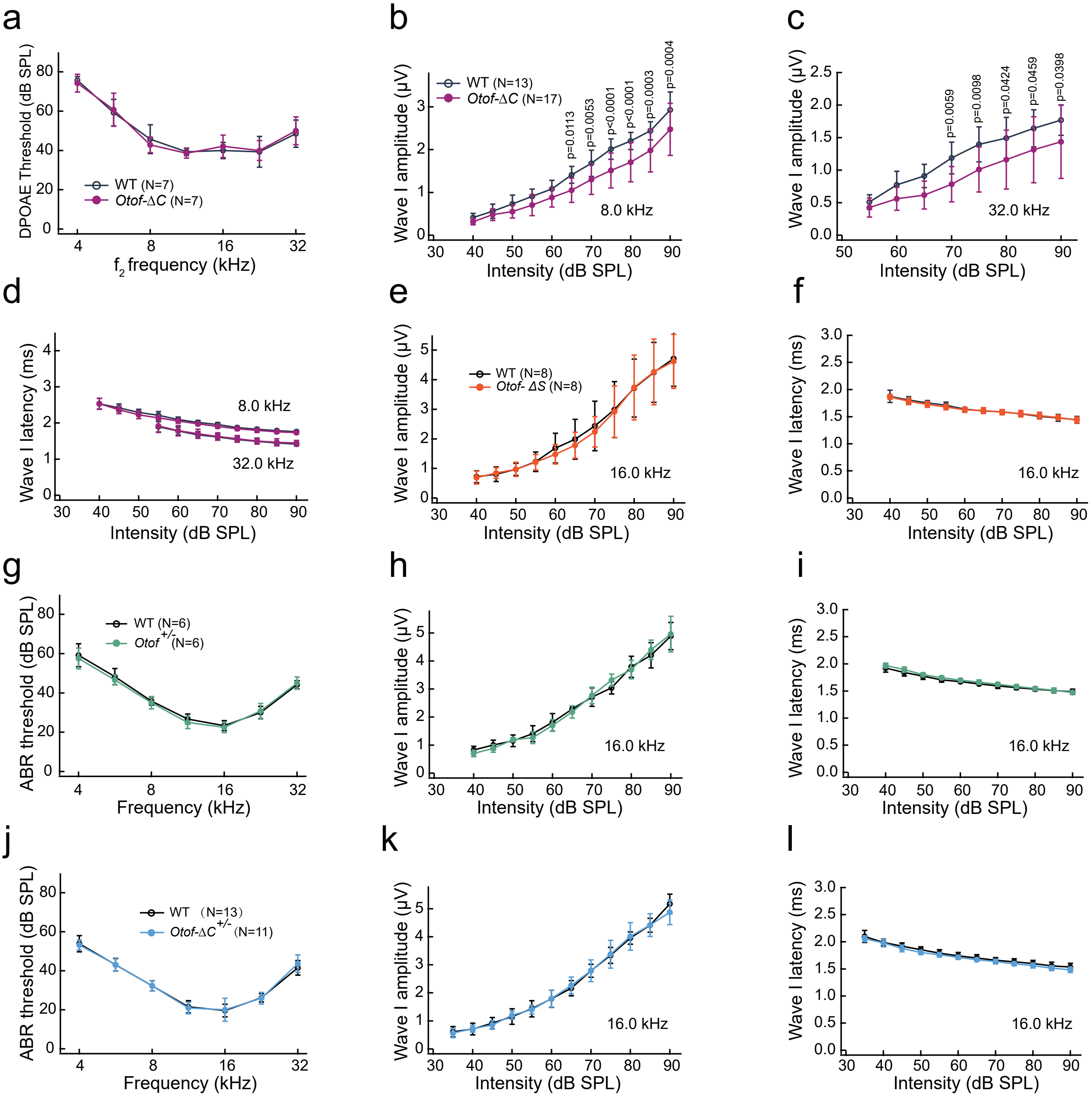

Supplementary Fig. 5 *Otof-ΔC* mice exhibited a decreased ABR wave I amplitude with normal latency, and the heterozygous *Otof-ΔC*, *Otof-ΔS* and *Otof*$^{-/-}$ mice show normal ABR.

a The DPOAE threshold was comparable between *Otof-ΔC* and WT mice. **b-d** ABR wave I amplitudes and latency are depicted between *Otof-ΔC* and WT mice. A significantly decreased wave I amplitude and normal latency was found at 8.0 and 32.0 kHz between the two animal models. **e, f** Audiograms show no differences between *Otof-ΔS* and WT mice. **g-i** The ABR threshold, wave I amplitude, and latency of *Otof*$^{+/-}$ mice virtually overlapped with results from WT mice, suggesting no detectable functional change under in vivo measurements. **j-l** Audiograms show no differences between WT and *Otof-ΔC*$^{+/-}$ mice. Data were analyzed by two-way ANOVA followed by the Bonferroni *post hoc* test. Age-matched and sex-matched littermate WT controls were used for all experiments. Statistical analysis by two-way ANOVA followed by the Bonferroni *post hoc* test with significance indicated (**b, c**). All data, statistical test used and *p* values can be found in the source data file.

Supplementary Fig. 6

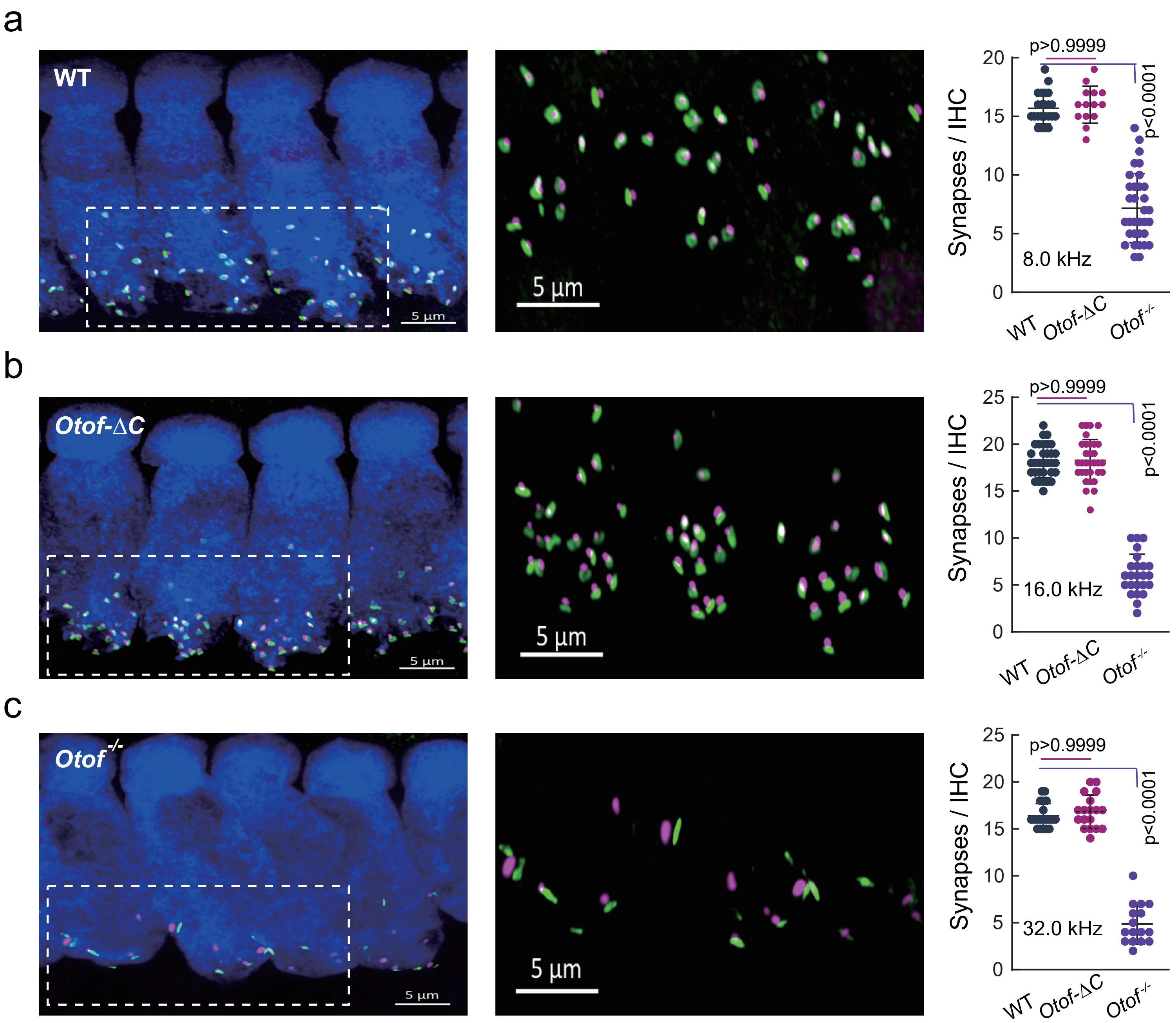

Supplementary Fig. 6 The inner hair cell ribbon synapse counts.

a-c Inner hair cell (IHC) ribbon synapse counts in mice with three different genotypes in three selected frequency regions of the cochlea. Left and middle: Confocal microscopy images of IHCs labeled with myosin VIIa (blue), the presynaptic marker CtBP2 (purple) and the postsynaptic marker GluA2 (green). Right: The number of co-labeled puncta representing functional ribbon synapses per IHC was counted. From apical to basal turns of the basilar membrane, the number of ribbon synapses per IHC was comparable between WT and *Otof-ΔC* mice, while the *Otof* $^{-/-}$ mice showed a significant reduction in the number of ribbon synapses. The data were analyzed by one-way ANOVA followed by the Bonferroni *post hoc* test. Age-matched and sex-matched littermate WT controls were used for all experiments. Statistical analysis by one-way ANOVA followed by the Bonferroni *post hoc* test with significance indicated (**a-c**). All data, statistical test used and *p* values can be found in the source data file.

Supplementary Fig. 7

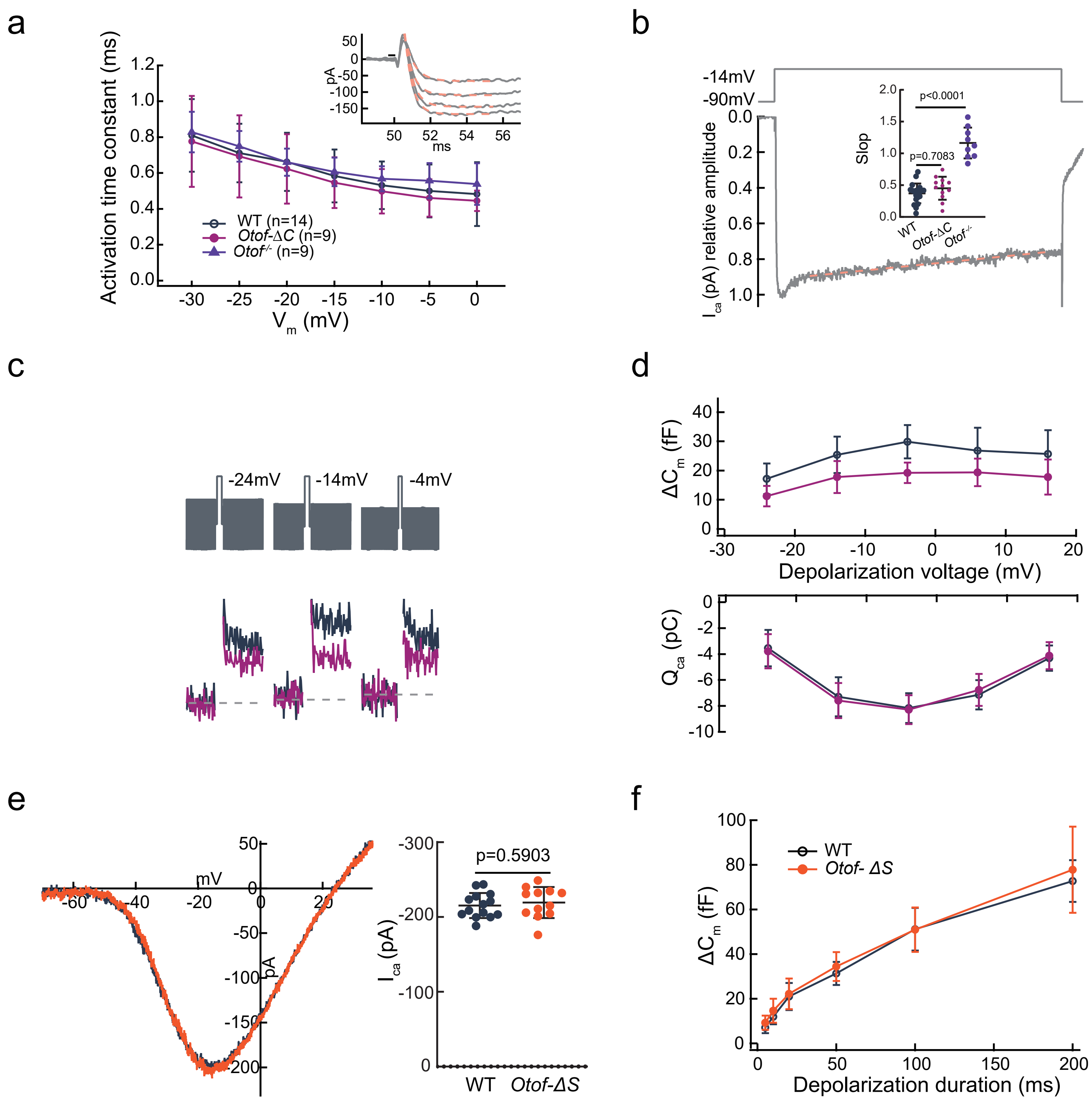

Supplementary Fig. 7 *Otof-ΔC* IHCs showed normal Ca^{2+} influx but reduced presynaptic exocytosis under different depolarization amplitudes.

a Activation time constants of Ca^{2+} currents at different depolarization potentials were obtained using a single exponential fit to the first 5 ms of the current traces, and no differences among all three groups were observed. **b** Average peak-normalized Ca^{2+} currents and Ca^{2+} current inactivation (a linear function fitted current) showed a faster decay of I_{Ca} in *Otof* $^{-/-}$ IHCs, whereas the inactivation of I_{Ca} was indistinguishable between *Otof-ΔC* and WT IHCs. **c** Representative ΔC_m traces in response to 50 ms step-depolarizations to −24, –14 and −4 mV, respectively. **d** The plot of ΔC_m versus voltage responses measured at a depolarization time of 50 ms showed a significant reduction in exocytosis in *Otof-ΔC* IHCs without alternations in calcium influx. **e** The Ca^{2+} current-voltage curves

showed comparable Ca^{2+} currents in *Otof-ΔS* and WT IHCs. **f** ΔC_m evoked by depolarizing pulses with durations ranging from 5 to 200 ms, and significant differences were found between *Otof-ΔS* and WT mice. Age-matched and sex-matched littermate WT controls were used for all experiments. Statistical analysis by one-way ANOVA followed by the Bonferroni *post hoc* test with significance indicated (**b**), one-way ANOVA followed by the Bonferroni *post hoc* test with significance indicated (**d**). All data, statistical test used and *p* values can be found in the source data file.

Supplementary Fig. 8

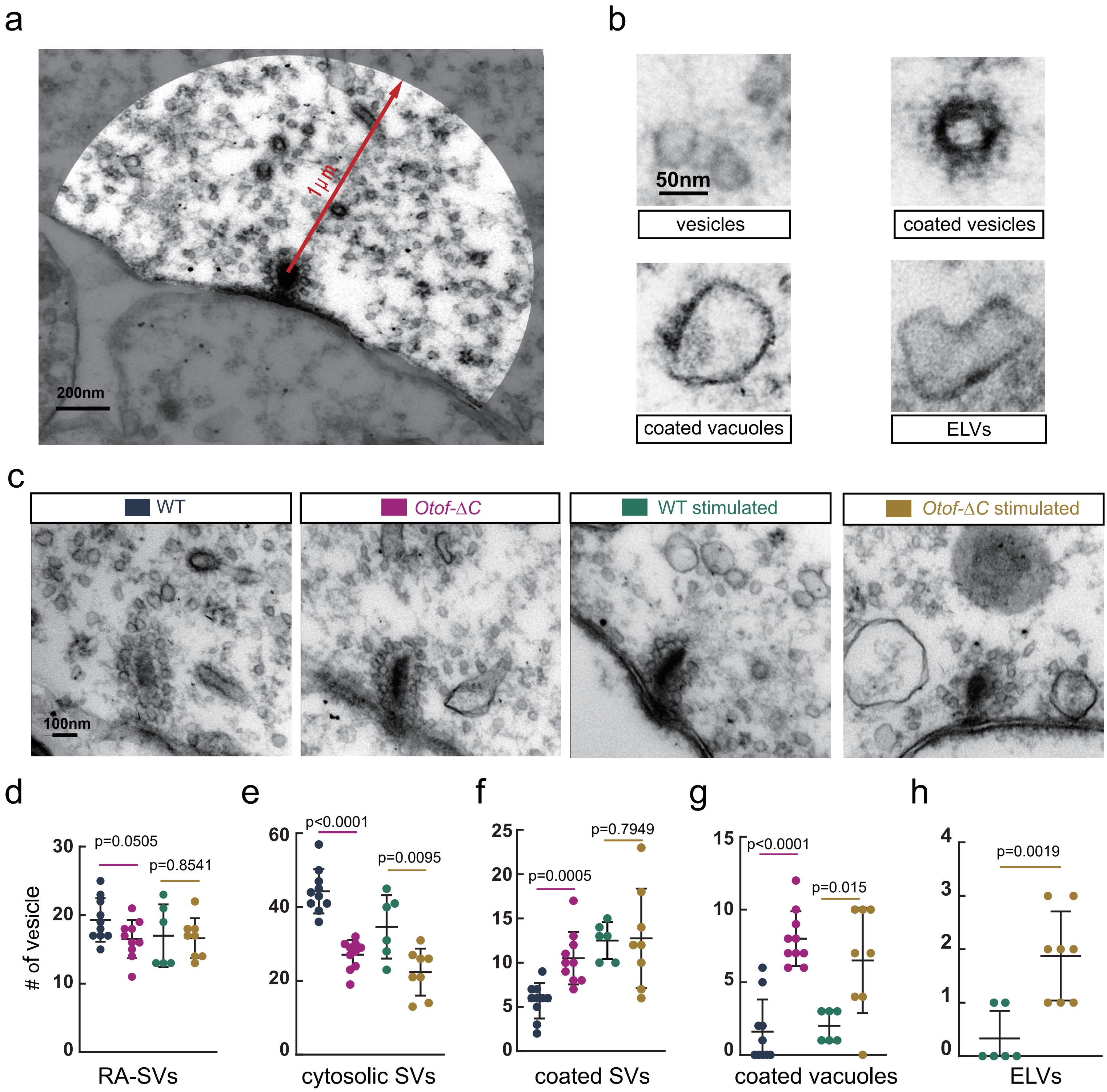

Supplementary Fig. 8 Decreased cytosolic synaptic vesicles, accumulation of coated structures, and ELVs in Otof-ΔC IHCs.
a A representative region of interest for analyzing ribbon synapses within a radius of r = 1 μm from the ribbon center in transmission electron micrographs (representative results of at least three biological replicates are shown). **b** The structural components in the ribbon synapse that were further quantified (**d-h**). Representative results of at least three biological replicates are shown. **c** Electron micrographs of ribbon synapses under resting and K^+-stimulated (40 mM for 5 min) conditions revealed changes in endocytic membrane recycling before and after depolarization-triggered vesicle release (representative results of at least three biological replicates are shown). **d, e** The number of ribbon-associated synaptic vesicles (RA-SVs, the first layer of SVs around the ribbon) was comparable between the two groups under both conditions (**d**). In contrast, the number of cytosolic synaptic vesicles was significantly reduced in *Otof-ΔC* IHCs (**e**). **f, g** The numbers of clathrin-coated structures (coated SVs and coated vacuoles) were significantly increased in the resting and recovery phases after neuronal stimulation in *Otof-ΔC* IHCs, suggesting deficits in sustained exocytosis. (**h**) The number of endosome-like vacuoles (ELVs) increased greatly after stimulation in *Otof-ΔC* IHCs, indicating the impairment of endocytic membrane retrieval and reformation. Age-matched and sex-matched littermate WT controls were used for all experiments. Statistical analysis by two-side unpaired *t* test or Mann-Whitney test with significance indicated (**d-h**). All data, statistical test used and *p* values can be found in the source data file.

Supplementary Fig. 9

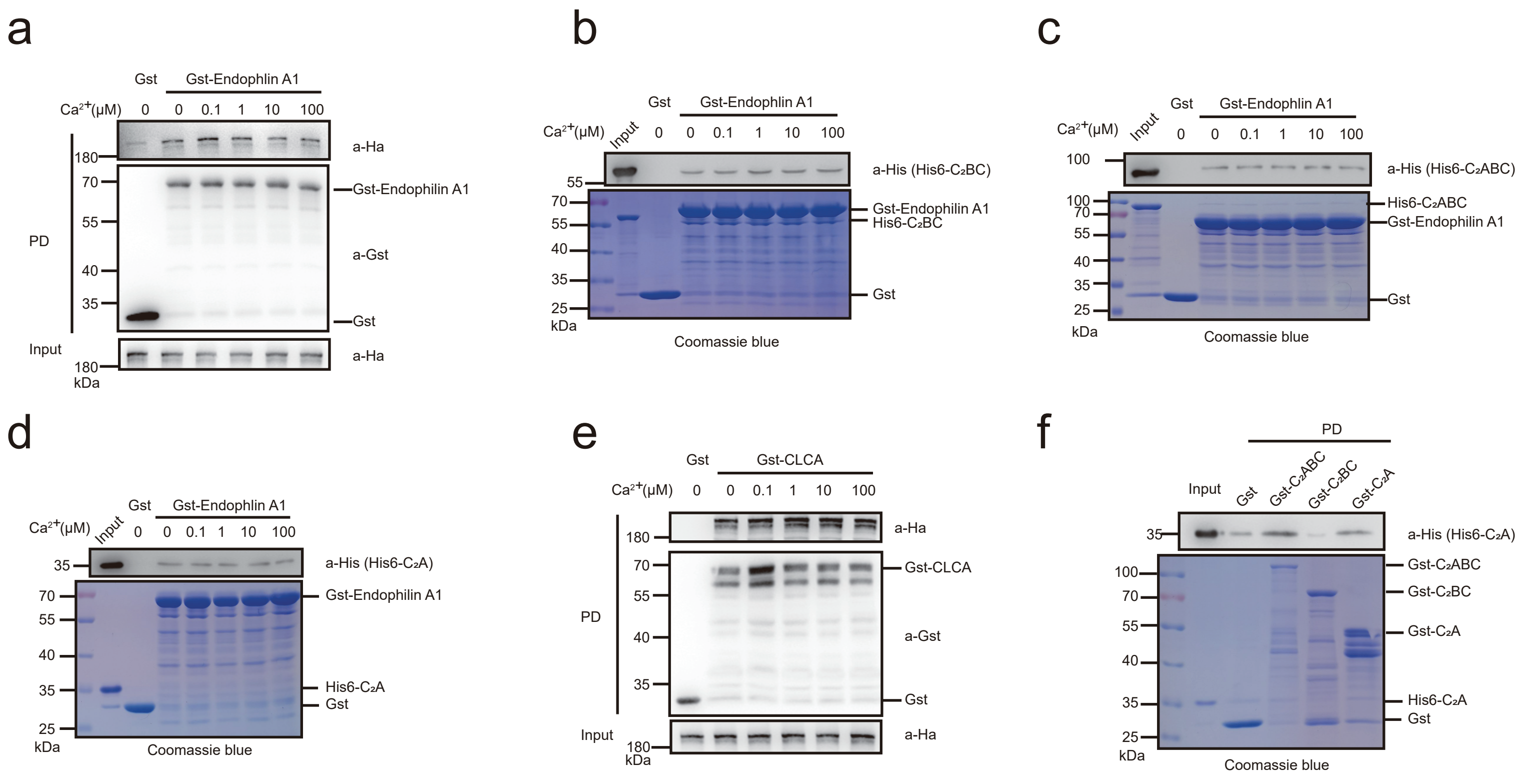

Supplementary Fig. 9 Structure of the otoferlin canonical and short isoform and protein binding assay.
a-e Effect of Ca^{2+} on otoferlin and its fragments (C_2ABC, C_2BC, and C_2A) binding with endophilin A1 or CLCA in the GST pull-down assay, and no obvious changes in the binding ability were found under different Ca^{2+} concentration. **f** Binding of His6-tagged C_2A to GST control and GST-C_2ABC, GST-C_2BC, and GST-C_2A in the GST pull-down assay. Otoferlin fragments that contain the C_2A domain exhibit a higher self-binding ability. Representative results of at least three biological replicates are shown.
